# Mask use in Chinese children admitted to the outpatient department: a single-center cross-sectional study

**DOI:** 10.1265/ehpm.24-00106

**Published:** 2024-11-02

**Authors:** Qian Yang, Jin Yu Chen, Qi Jiang, Yan Fang Zhang, Dao Ting Li, Cai Yun Xia, Ying Cai, Man Man Niu, Jin Wei Ruan, Peng Hu

**Affiliations:** Department of Paediatrics, the First Affiliated Hospital of Anhui Medical University, No. 218 Ji-Xi Road, Hefei, Anhui Province, 230022, PR China

**Keywords:** Adverse events, Children, Coronavirus disease-2019, Mask

## Abstract

**Background:**

Mask use is a critical precaution to prevent the transmission of SARS-CoV-2 in a crowded or densely populated indoor environment. There is still a lack of large-sample studies on mask use in children during the COVID-19 pandemic.

**Methods:**

A questionnaire was distributed to individuals under 18 years of age from the pediatric outpatient department from November 2021 to May 2022. Participants who were willing to be interviewed and had good communication and judgment skills participated in our study.

**Results:**

5053 (a boy-to-girl ratio of 1.13:1 and a median age of 5 years) from 6200 individuals admitted to the pediatric outpatient department were enrolled in the study. The mask-wearing time increased in parallel with age. Children aged 3–5 years wore masks more correctly (*χ*^2^ = 41.591, *P* < 0.05), complained more about the discomfort (*χ*^2^ = 193.871, *P* < 0.05), and their parents/caregivers were significantly better aware of the preventive effect of masks on respiratory disease (*χ*^2^ = 19.501, *P* < 0.05) than parents/caregivers of other age groups. Masks designed for children were more used by those aged 3–5 years in outdoor settings. The commonest adverse events of mask-wearing were respiratory symptoms (61.2%), followed by dermatological symptoms (28.9%) and psychological symptoms (19.7%). Girls wore masks for a longer time and more correctly (*χ*^2^ = 10.598, *P* < 0.05) than boys. Compared with the pre-COVID-19 pandemic, wearing masks could significantly decrease the median frequency of respiratory infections during the COVID-19 pandemic (2[1–4] *vs* 3[2–4]; z = −2.692, *P* < 0.05).

**Conclusions:**

Wearing proper and well-fitted masks could significantly protect children from respiratory infections in a crowded or densely populated indoor environment during the COVID-19 pandemic. However, mask-associated adverse events, particularly in psychological symptoms, are needed to draw adequate attention, calling for early identifications and psychological interventions.

**Supplementary information:**

The online version contains supplementary material available at https://doi.org/10.1265/ehpm.24-00106.

## Introduction

Coronavirus disease 2019 (COVID-19), caused by severe acute respiratory syndrome coronavirus 2 (SARS-CoV-2) infection, has imposed a significant global public health burden since its first discovery in December 2019 [1]. According to the guidelines issued by the World Health Organization (WHO), SARS-CoV-2 is primarily transmitted between people through respiratory droplets and direct or indirect contact with contaminated surfaces [2].

Mask use has become an important omnipresent precaution to prevent the transmission of SARS-CoV-2 in a crowded or densely populated indoor environment [3]. A survey from Kansas revealed a decline in cases among counties with a mask mandate, whereas a persistent increase in cases in counties without a mask mandate (0.08 versus 0.11 cases per 100,000 per day) [4]. Nonetheless, wearing masks in public has not always been embraced by the public, and attitudes toward mask use vary noticeably among different ethnicities. A recent online recruitment survey showed that 87.5% of Chinese respondents held favorable attitudes toward mask wearers, compared to 52% of non-East Asian Canadian respondents [5]. Respondents reporting higher levels of trust in information from government sources are more likely to wear masks, despite that a series of adverse events have been reported in adults, such as mask-related acne and irritant contact dermatitis [6]. However, there is a lack of large-sample studies on mask use in children during the COVID-19 pandemic. On this background, the present study investigated the mask use in children admitted to the outpatient department, aiming to help children wear masks correctly and reduce the risk of nosocomial infection.

## Methods

### Participants

The study population comprised individuals under 18 years of age from the pediatric outpatient department. A 14-item questionnaire (Supplementary questionnaire) was developed following the advice on the use of masks guideline issued by the World Health Organization [2]. For each child recruited in the present study, informed consent was obtained from available caregivers. Exempt for the present study was acquired from the Medical Ethic Committee of the First Affiliated Hospital of Anhui Medical University.

### Data collection

This cross-sectional survey was conducted from November 2021 to May 2022 in Anhui, China. All questionnaires were conducted during the COVID-19 pandemic. The inclusion criteria were participants (1) admitted to our pediatric outpatient clinic, (2) willing to be interviewed, and (3) having good communication and judgment skills. The key exclusion criteria were (1) neonates, (2) refusing to participate in this study, (3) unwilling to wear masks in a crowded or densely populated indoor environment, (4) caregivers unfamiliar with their kids, and (5) individuals with a pre-existing history of skin, oral, cardiovascular and neurological problems before mask use. We showed the proper/correct manner of wearing masks to parents/caregivers by watching a video (https://v.qq.com/x/page/x3345dei7ef.html).

### Statistical analysis

SPSS 22.0 was used for statistical analysis. Continuous data were expressed as median (Interquartile range: IQR) and analyzed using Mann-Whitney U test or Kruskal-Wallis H Test. Categorical data were presented as numbers/percentages and compared using Chi-square test. All *P* values were 2-sided and *P* < 0.05 was considered statistically significant.

## Results

### Question 1–2: demographic data

**Fig. 1:**
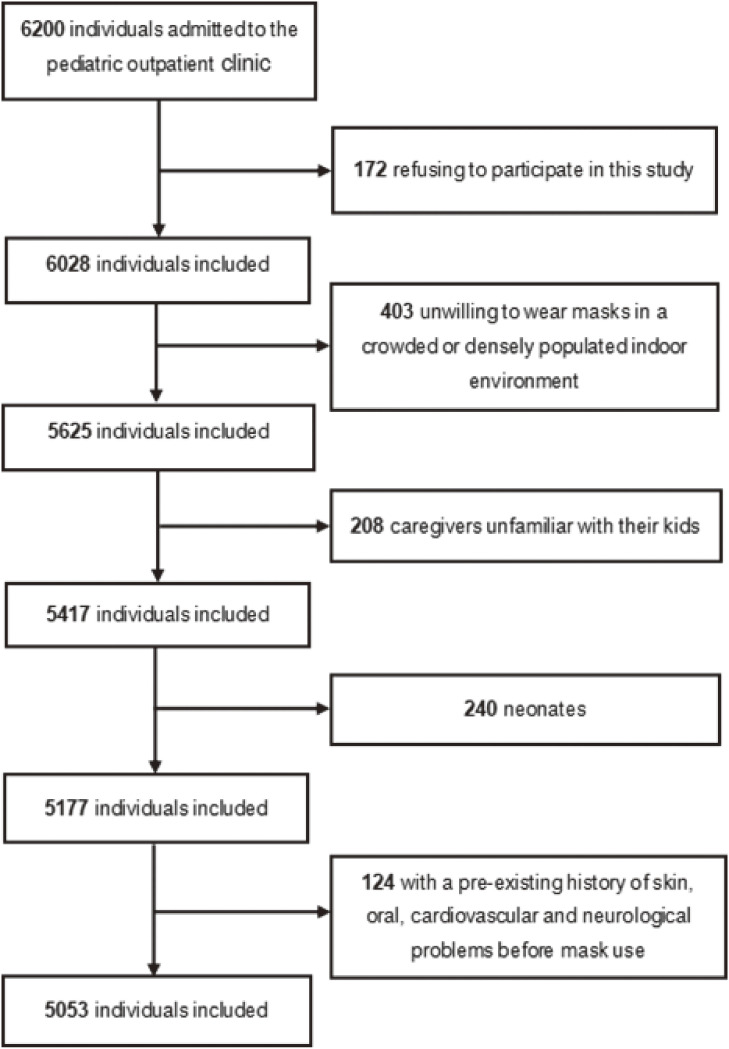
Flowchart of participant selection

A flowchart of the participants is shown in Fig. 1. A total of 6200 individuals admitted to the pediatric outpatient department were invited to participate in this survey. Of these individuals, 172 refused to participate in this survey, 403 were excluded for being unwilling to wear masks in a crowded or densely populated indoor environment, and 208 were excluded because caregivers were unfamiliar with their kids. 240 neonates were excluded for being unsuitable to wear masks and 124 individuals were excluded with a pre-existing history of skin, oral, cardiovascular and neurological problems before mask use. Finally, 5053 participants were enrolled in the subsequent analyses, with a boy-to-girl ratio of 1.13:1 (2680:2373) and a median (IQR) age of 5 (3–7) years. In this study, 49 (1.0%) children were under 1 year of age, 655 (13.0%) aged 1–2 years, 2408 (47.7%) aged 3–5 years, 1731 (34.3%) aged 6–11 years, and 210 (4.2%) aged 12–17 years.

### Question 3: mask types

Detailed characteristics of mask types among age groups and between genders are shown in Table 1. 4151 participants (82.1%) wore masks designed for children, 521 (10.3%) wore surgical masks, 19 participants (0.4%) wore N95 masks, and 362 participants (7.2%) used multiple masks occasionally. Among 4151 participants wearing the specially designed masks for children, 47 (1.1%) were under 1 year of age, 606 (14.6%) aged 1–2 years, 2147 (51.7%) aged 3–5 years, 1326 (32.0%) aged 6–11 years, and 25 (0.6%) aged 12–17 years. Among 521 participants wearing surgical masks, 19 (3.6%) aged 1–2 years, 65 (12.5%) aged 3–5 years, 262 (50.3%) aged 6–11 years, and 175 (33.6%) aged 12–17 years. Among 19 participants wearing N95 masks, 7 (36.9%) aged 3–5 years, 10 (52.6%) aged 6–11 years, and 2 (10.5%) aged 12–17 years. The mask types were significantly different among age groups (*χ*^2^ = 1476.213, *P* < 0.05). Masks designed for children were more used by children aged 3–5 years, and surgical masks were more used by children aged 6–11 years. However, no significant difference was found in mask types between genders (*χ*^2^ = 7.706, *P* > 0.05).

**Table 1:** The results of questionnaire survey on mask use among age groups and between genders

	**Total** **(n = 5053)**	**Age groups**					***P* value**	**Genders**		***P* value**
	
		**Under 1 year ** **of age** **(n = 49)**	**1–2 years** **(n = 655)**	**3–5 years** **(n = 2408)**	**6–11 years** **(n = 1731)**	**12–17 years** **(n = 210)**		**Boys** **(n = 2680)**	**Girls** **(n = 2373)**	
**Mask types**							**<0.001**			0.052
Mask specially designed for children, n (%)	4151(82.1%)	47(95.9%)	606(92.5%)	2147(89.2%)	1326(76.6%)	25(12%)		2168(80.9%)	1983(83.5%)	
Surgical mask, n (%)	521(10.3%)	0	19(2.9%)	65(2.7%)	262(15.1%)	175(83.3%)		305(11.4%)	216(9.1%)	
N95 mask, n (%)	19(0.4%)	0	0	7(0.3%)	10(0.6%)	2(1.0%)		10(0.4%)	9(0.4%)	
Multiple masks, n (%)	362(7.2%)	2(4.1%)	30(4.6%)	189(7.8%)	133(7.7%)	8(3.8%)		197(7.3%)	165(7.0%)	
**Mask-wearing time, median (IQR), hour**										
Average daily mask use	1(0.5–1)	0.5	0.5	0.5(0.5–1)	1(1–2)	2(1–5)	**<0.001**	1(0.5–1)	1(0.5–1)	0.191
Maximum duration of mask use	3(2–4)	1(0.5–1.5)	1.5(0.5–3)	3(2–4)	4(3–4)	4(4–5)	**<0.001**	3(2–4)	3(2–4)	**<0.001**
Mask replacement cycle	5(3–24)	1(0.5–2)	3(1–24)	4(3–24)	24(4–24)	24(4–24)	**<0.001**	4(3–24)	7(3–24)	**<0.001**
**Circumstances of mask use**							**<0.001**			0.259
Indoor settings, n (%)	5053(100%)	49(100%)	655(100%)	2408(100%)	1731(100%)	210(100%)		2680(100%)	2373(100%)	
Outdoor settings, n (%)	2965(58.7%)	8(16.3%)	309(47.2%)	1327(55.1%)	1160(67%)	161(76.7%)		1506(56.2%)	1459(61.5%)	
**Mask-wearing behaviors**							**<0.001**			**0.026**
Proper, n (%)	3290(65.1%)	40(81.6%)	445(67.9%)	1488(61.8%)	1153(66.6%)	164(78.1%)		1768(66.0%)	1522(64.1%)	
Improper, n (%)	1763(34.9%)	9(18.4%)	210(32.1%)	920(38.2%)	578(33.4%)	46(21.9%)		912(34.0%)	851(35.9%)	
Wear masks only covering mouths, n (%)	1372(27.2%)	8(16.4%)	170(26.0%)	700(29.1%)	460(26.6%)	34(16.2%)		684(25.5%)	688(29.0%)	
Wear masks not covering noses and mouths, n (%)	391(7.7%)	1(2.0%)	40(6.1%)	220(9.1%)	118(6.8%)	12(5.7%)		228(8.5%)	163(6.9%)	
Wear masks that do not fit, n (%)	918(18.2%)	5(10.2%)	116(17.7%)	527(21.9%)	244(14.1%)	26(12.4%)		485(18.1%)	433(18.2%)	
Reuse masks, n (%)	755(14.9%)	3(6.1%)	74(11.3%)	377(15.7%)	276(16.0%)	25(11.9%)		371(13.8%)	384(16.2%)	
Wear the opposite side of masks, n (%)	29(0.6%)	0	4(0.6%)	10(0.4%)	14(0.8%)	1(0.5%)		17(0.6%)	12(0.5%)	
Wear others’ masks by mistake	5(0.1%)	0	0	4(0.2%)	1(0.1%)	0		2(0.1%)	3(0.1%)	
**Did your child complain about the adverse events of mask wearing?**							**<0.001**			0.103
Yes, n (%)	2307(45.7%)	8(16.3%)	237(36.2%)	1191(49.5%)	808(46.7%)	63(30.0%)		1178(44.0%)	1129(47.6%)	
No, n (%)	2746(54.3%)	41(83.7%)	418(63.8%)	1217(50.5%)	923(53.3%)	147(70.0%)		1502(56.0%)	1244(52.4%)	
**Did masks have a preventive effect on respiratory disease?**							**<0.001**			0.055
Yes, n (%)	4759(94.2%)	49(100.0%)	627(95.0%)	2240(93.0%)	1636(94.5%)	207(98.6%)		2540(94.8%)	2219(93.5%)	
No, n (%)	294(5.8%)	0	28(5.0%)	168(7.0%)	95(5.5%)	3(1.4%)		140(5.2%)	154(6.5%)	
**Did the frequency of respiratory infections decrease during the COVID-19 period compared with the pre-COVID-19 period?**							**<0.001**			0.545
Yes, n (%)	1849(36.6%)	2(4.4%)	138(21.1%)	780(32.4%)	819(47.3%)	110(52.4%)		991(37.0%)	858(36.2%)	
No, n (%)	3204(63.4%)	47(95.6%)	517(78.9%)	1628(67.6%)	912(52.7%)	100(47.6%)		1689(63.0%)	1515(63.8%)	

Note: Boldface indicates statistical significance (*P* < 0.05)

Abbreviations: COVID-19, coronavirus disease 2019; IQR, interquartile range.

### Question 4–6: mask-wearing time

Detailed characteristics of mask-wearing time among age groups and between genders are shown in Table 1. The median (IQR) time of average daily mask use was 1 (0.5–1) hour among 5053 children, with half an hour in children under 6 years of age, 1 (1–2) hour in children aged 6–11 years and 2 (1–5) hours in children aged 12–17 years, respectively. The median (IQR) time of maximum duration of mask use was 3 (2–4) hours among 5053 children, with 1 (0.5–1.5) hour in children under 1 year of age, 1.5 (0.5–3) hours in children aged 1–2 years, 3 (2–4) hours in children aged 3–5 years, 4 (3–4) hours in children aged 6–11 years, and 4 (4–5) hours in children aged 12–17 years. The median (IQR) time of the mask replacement cycle was 5 (3–24) hours among 5053 children, with 1 (0.5–2) hour in children under 1 year of age, 3 (1–24) hours in children aged 1–2 years, 4 (3–24) hours in children aged 3–5 years, 24 (4–24) hours in children aged 6–17 years. The mask-wearing time was significantly different among age groups (*P* < 0.05). The mask-wearing time increased in parallel with age. In addition, the time of maximum duration of the wearing and mask replacement cycle was significantly different between genders (*P* < 0.05). Girls wore masks for a longer duration and had a longer replacement cycle than boys.

### Question 7: circumstances of mask use

Detailed characteristics of circumstances of mask use among age groups and between genders are shown in Table 1. 11 children (0.2%) wore masks in childcare centers, 403 (8.0%) in schools, 4944 (97.8%) in malls, 4941 (97.8%) when they took public transportation, 5050 (99.9%) in hospitals, and 2965 (58.7%) in outdoor settings. The circumstances of mask use were significantly different among age groups (*χ*^2^ = 2097.766, *P* < 0.05). Masks were more used by children aged 3–5 years in outdoor settings. However, no significant difference was found in circumstances of mask use between genders (*χ*^2^ = 43.655, *P* > 0.05).

### Question 8, 11: mask-wearing behaviors

Detailed characteristics of the mask-wearing behaviors among age groups and between genders are shown in Table 1. 3290 (65.1%) children wore masks covering their noses and mouths, 1372 (27.2%) wore masks only covering their mouths, 391 (7.7%) wore masks not covering their noses and mouths but pulling down the masks under their chins, 918 (18.2%) wore masks that do not fit, 755 (14.9%) reused masks, 29 (0.6%) wore the opposite side of masks, and 5 (0.1%) wore others’ masks by mistake. The age group distributions of 3290 children who wore masks correctly were 40 (1.2%) under 1 year of age, 445 (13.5%) aged 1–2 years, 1488 (45.2%) aged 3–5 years, 1153 (35.1%) aged 6–11 years, and 164 (5.0%) aged 12–17 years. The mask-wearing behaviors were significantly different among age groups (*χ*^2^ = 41.591, *P* < 0.05) and between genders (*χ*^2^ = 10.598, *P* < 0.05).

### Question 9–10: adverse events of mask-wearing

Adverse events of mask-wearing are shown in Table 2. 2307 participants had complained about the adverse events of mask-wearing. Of them, 1412 (61.2%) reported respiratory symptoms, 666 (28.9%) reported dermatological symptoms, 454 (19.7%) reported psychological symptoms and 390 (16.9%) reported other symptoms including hot and vision impairment. Detailed data on the adverse events of mask-wearing among age groups and between genders are shown in Table 1. Of the 2307 children complaining about the adverse events of mask-wearing, 8 (0.4%) were under 1 year of age, 237 (10.3%) aged 1–2 years, 1191 (51.6%) aged 3–5 years, 808 (35.0%) aged 6–11 years, and 63 (2.7%) aged 12–17 years. The adverse events of mask-wearing were significantly different among age groups (*χ*^2^ = 193.871, *P* < 0.05), whereas not significantly different between genders (*χ*^2^ = 5.210, *P* > 0.05). 1461 participants (68.1%) complained about the adverse events of masks in both quiet and active conditions, 211 (9.8%) in quiet conditions and 475 (22.1%) in active conditions.

**Table 2:** The adverse events of mask wearing

**Symptoms**	**Number**	**Percentage**
**Respiratory symptoms**		
Dyspnea	1387	60.12%
Chest distress	19	0.82%
Itchy throat	2	0.09%
Cough	1	0.04%
Nasal congestion	1	0.04%
Sneeze	1	0.04%
Runny nose	1	0.04%
**Dermatological symptoms**		
Tightness/pressure	369	15.99%
Itching	89	3.86%
Allergic dermatitis	8	0.35%
Redness	3	0.13%
Broken	1	0.04%
**Psychological symptoms**		
Unhappy	320	13.87%
Not communicating with others	224	9.71%
Inattentive	77	3.34%
Irritable	61	2.64%
Decreased learning efficiency	3	0.13%
Anxiety or fear of going out	2	0.09%
Have nightmares	1	0.04%
Don’t want to go to school	1	0.04%
**Other symptoms**		
Hot	273	11.83%
Moist mask	196	8.50%
Vision impairment	106	0.26%
Unpleasant smell of mask	6	0.26%
Dry mouth	3	0.13%
Headache	2	0.09%

### Question 12–14: preventive effect of masks on respiratory disease

Detailed data on the preventive effect of masks on respiratory disease among age groups and between genders are shown in Table 1. A total of 4759 participants considered that masks had a preventive effect on respiratory disease. Compared with the pre-COVID-19 pandemic, there was a significant decrease in the median (IQR) frequency of respiratory infections during the COVID-19 pandemic (2 [1-4] *vs* 3 [2-4]; *z* = −2.692, *P* < 0.05). Of them, 49 (1.0%) were under 1 year of age, 627 (13.2%) aged 1–2 years, 2240 (47.1%) aged 3–5 years, 1636 (34.4%) aged 6–11 years, and 207 (4.3%) aged 12–17 years. The preventive effect of masks on respiratory disease was significantly different among age groups (*χ*^2^ = 19.501, *P* < 0.05). Caregivers of children aged 3–5 years were significantly better aware of the preventive effect of masks on respiratory disease than the others. However, no significant difference was found in the preventive effect of masks between genders (*χ*^2^ = 3.680, *P* > 0.05).

## Discussion

According to the WHO guidelines, children should wear well-fitted disposable medical masks in indoor settings where ventilation is poor or unknown, and an N95 mask or a medical mask is recommended to be used in medical institutions [7, 8]. However, it’s not needed for children to wear a mask when doing physical activities or in sparsely populated outdoor settings [7]. In the present study, we interviewed the caregivers of 5053 children and found that 82.1% of them wore masks designed for children in indoor or outdoor settings, 10.3% wore surgical masks and only 0.4% wore N95 masks in hospitals. Similarly, a recent study conducted in Thailand discovered that 52.3% of participants wore fabric masks, 44.8% wore surgical masks and only 2.8% wore N95 masks routinely [9]. The paucity of children wore N95 masks during the COVID-19 pandemic, which may be caused by the dearth of N95 masks made designed for children; on the other hand, lack of awareness of the proper wearing method for N95 masks may be considered as another important reason [9, 10]. Despite the fact that the majority of caregivers chose masks designed for children in our study, there is currently no global standard for children’s masks. On the basis of the expert consensus by the State Administration for Market Regulation and the Standardization Administration of China, children’s masks fulfilling national standard GB/T 38880-2020 are just recommended for these children aged 6–14 years [11]. On this background, an improved standard of children’s masks should be warranted to be established for all age children.

To elucidate the impact of age on mask-wearing, our study investigated, for the first time, mask-wearing time among age groups and found that mask-wearing time was significantly increased in parallel with age. Likewise, Haischer *et al.* [12] conducted a direct observational study including 9935 shoppers in Wisconsin between June and August 2020, and found that the percentage of wearing masks was 37% in young individuals, 41% in the middle-aged and 57% in the older, respectively; the odds of individuals wearing masks increased significantly with age and was also 1.5× greater for females than males. Two possible reasons for the age-associated trend were the wider activity sphere of the older children than the younger, and the increasing opportunities for older children to stay in places where wearing masks were needed, such as crowded classrooms or public transportation. Since September 2020, wearing masks in school was no longer a mandatory requirement and the daily use time of masks decreased from 7–8 hours to no more than 2 hours to maintain the physical and mental health of students. In the present study, the observation period was located in the period from November 2021 to May 2022, thus the average daily use time was no more than 2 hours in children aged 6–17 years. In addition, girls in our study wore masks for a longer duration and had a longer replacement cycle than boys, which may be explained by the gender discrepancies in personality traits. Slobodskaya *et al.* [13] measured the personality of Russian children in two communities by the Inventory of Child Individual Differences-Short version, and noted that boys tended to score 1 T-score higher on activity (a component of extraversion) than girls. An active and energetic boy appears prone to taking off the mask when feeling any discomfort, which may lead to the shorter mask-wearing time and more frequent replacement cycle.

A poor-fitted or uncomfortable mask tends to be worn incorrectly or fall down. The gaps between the mask and the face allow air with respiratory droplets to leak in and out around the edges of the mask, causing the transmission of pathogens [10]. According to the American Academy of Pediatrics guideline, children who wear masks covering the mouth and nose are deemed correct [14]. In the present study, the rate of children who wore masks incorrectly was 7.8%, which was slightly higher than a recent study by Haischer *et al.* [12], in which 6% of participants wore masks incorrectly before store mandates. Furthermore, our findings also suggested that 14.9% of participants reused a same mask more than two times on different occasions, yielding a more potential risk of SARS-CoV-2 transmission [15]. Nguyen *et al.* [16] reported that healthcare workers reusing masks were related to a 46% increased risk of a positive SARS-CoV-2 test compared to those not. Interestingly, children aged 3–5 years were found to show more correctness of mask use in our study, since a more assistance from their caregivers than the other age groups. Taking the particularity of children’s growth and development into account, a broad mask-wearing education should be launched to reinforce good hygiene practices in appropriate manners welcomed by children such as cartoon videos or story boxes [17].

Although mask-wearing acts as an effective measure to reduce the spread of SARS-CoV-2 by cutting off the droplet transmission route directly, the use of masks can also lead to some adverse events in children. In the present study, respiratory symptoms were the commonest adverse events of mask-wearing and occurred in 61.2% of children, followed by dermatological symptoms (28.9%) and psychological symptoms (19.7%). Farronato *et al.* [18] recruited 256 Italian dentists wearing masks and revealed 63.5% of them reported moderate breathing difficulties, 54.3% reported moderate concentration problems, 50.8% reported severe exertion and 47.5% reported headaches. It should be worth noting that a subset of participants underwent psychological symptoms both in our study and the others. A recent observational study encompassing 1,199,320 students showed that psychological distress was self-reported by 10.5% of participants, and moreover, the risk for psychological distress of individuals who never wore a mask experienced a 2.64-fold higher than those who always wore a mask, owing to the increased anxiety on the numbers of COVID-19 cases and deaths [19]. On the contrary, the present study revealed that psychological symptoms also occurred in 19.7% of participants who wore a mask in indoor settings. We speculate that emotion recognition and social communication may be impaired by the long-term mask-wearing, and thereby result in psychological symptoms [20, 21].

Using a mask to control respiratory infections is a well-established strategy at the source. Compared with the pre-COVID-19 pandemic, the present study found a 33% decrease in the median frequency of respiratory infections during the COVID-19 pandemic. Leung *et al.* [22] analyzed the viral shedding of 111 participants with a respiratory infection, and indicated that the coronavirus, influenza virus and rhinovirus in respiratory droplet samples were reduced by 30%, 22% and 6% from participants wearing masks respectively, as compared with their counterparts. As for SARS-CoV-2, a recent meta-analysis encompassing 6 studies demonstrated the wide use of masks contributed to a 53% reduction in COVID-19 incidence [23], and moreover, a natural experiment across 200 countries showed 45.7% fewer COVID-19 related mortality in countries recommending masks [24]. Conversely, the lifting of masking requirements was associated with an additional 44.9 cases per 1000 students and staff during the 15 weeks after the statewide masking policy was rescinded in Massachusetts [25]. Therefore, a universal masking policy is critical to minimizing the inequitable harms caused by COVID-19 and maximizing our ability to learn, work, and socialize during the pandemic [26].

Despite the current evidence suggests a significant decrease in the overall risk of respiratory infections, our study showed that the majority of parents/caregivers of children younger than 12 years answered ‘No’ when we asked ‘Did the frequency of respiratory infections decrease during the COVID-19 period compared with the pre-COVID-19 period?’. We postulate that the discrepancy of which may be attributed to the following 3 reasons. First, based on the characteristics of children’s growth, children younger than 12 years have a relatively weak immunity [27]; in addition, due to the COVID-19 pandemic, the parents of children younger than 12 years became more anxious about the health of their offspring, which resulted in parents seeking medical helps more frequently when their offspring complained about even slight discomfort. Second, the majority of mask types were unsuitable for children younger than 12 years, associated with poor protection against respiratory infections [28]. Third, children younger than 12 years were reliant on their parents, and they were more likely to be infected once their parents had latent infections [29].

### Limitations and strengths

There are still several limitations in the present study. First, participants from a single hospital setting may cause selection bias, in spite of a large sample size. Second, the data from a cross-sectional study cannot be used to infer causal relationships. Third, the reliability of questionnaire survey results is greatly affected by parent reports. As some answers were based on the memories of parents/caregivers, the study results may be affected by recall bias. Fourth, some benign symptoms may be relieved spontaneously before an interview questionnaire, which leads to an underestimation of mask-associated adverse events. Fifth, it is difficult to distinguish whether some complaints are triggered by mask-wearing or other reasons, especially in those children receiving a recent vaccination.

Albeit with limitations, the present study also has some strengths. When we prepared the manuscript, seldom research was available regarding the impact of age on mask use in children during the COVID-19 pandemic. Our study investigated, for the first time, the mask-wearing time of children among age groups and found that mask-wearing time was significantly increased in parallel with age.

### Prospect

Currently, COVID-19 remains an established and ongoing health issue despite WHO announced it no longer constituted a public health emergency of international concern on 5 May 2023 [30]. Taking into account the importance of wearing masks to prevent and block the person-to-person transmission of SARS-CoV-2 and other respiratory viruses, we give some advice on children’s mask-wearing in the post-pandemic era. Parents/caregivers and teachers should recognize the importance of wearing masks. An appropriate type and well-fitted mask should be worn by children in areas where SARS-CoV-2 is spreading. Children with immunodeficiency or receiving immunosuppressors are recommended to wear medical masks in higher-risk situations year-round [31]; in contrast, those children with cognitive or respiratory impairments, developmental disorders, disabilities or other specific health conditions who experience difficulties are not required to wear masks [7]. As for health workers and visitors, N95 respirators/medical masks should be worn in healthcare facilities when they interact with children. Last but most important, the physical, psychological, cognitive, and communicative adverse events associated with mask-wearing are necessary to be given adequate attention and optimal intervention.

## Supplementary information

**Additional file 1:** Survey on mask use in children.

## Declarations

### Data availability statement

The datasets generated during and/or analyzed during the current study are available from the corresponding author on reasonable request.
