# Supplementary material for: Mask use in Chinese children admitted to the outpatient department: a single-center cross-sectional study
Source: Environ Health Prev Med. 2024 Nov 2;29:60. doi: 10.1265/ehpm.24-00106 (PMC11551440; doi:10.1265/ehpm.24-00106)
Supplement: Supplementary file 1 — Additional file 1: Survey on mask use in children. [file ehpm-29-060-s001.docx]

**Survey on mask use in children**

1. **The age of your child:** ( ) years old

**2. The gender of the child:**

① Boy ② Girl

1. **The type of mask your child wearing: (Multiple choice)**

① Mask designed for children ② Surgical mask ③ N95 mask

1. **The time of average daily mask use is:** ( ) hours
2. **The time of maximum duration of mask use is:** ( ) hours
3. **The time of the mask replacement cycle is:** ( ) hours
4. **The circumstances of mask use: (Multiple choice)**
5. Childcare centers ② School ③ Mall ④ Public transportation

⑤ Hospital ⑥Outdoors

1. **How did your child wear the mask:**

① Covering nose and mouth ② Covering mouth ③ Not covering noses and mouths but pulled down the masks under chins

1. **The complaints about mask wearing in the child: (Multiple choice)**
2. No ② Headache ③ Fever ④ Fatigue ⑤ Tachycardia ⑥ Chest tightness

⑦ Dyspnea ⑧ Sore throat ⑨ Cough ⑩ Sneeze ⑪ Skin redness ⑫ Skin itching

⑬ Skin broken ⑭ Acne ⑮ Drowsiness ⑯ Vertigo ⑰ Vision impairment

⑱ Dry mouth ⑲ Runny nose ⑳ Other: [free text]

1. **The condition when the above complaints occurred:**

① In quiet condition (Keep a physical or behavioral rest state) ② In active condition (Carry out physical activities) ③ In both quiet and active condition

1. **The improper mask-wearing behavior observed in the child: (Multiple choice)**
2. Wore others’ masks by mistake ② Wore the opposite side of masks ③ Not fit

④ Reuse ⑤ None

1. **Did masks have a preventive effect on children's respiratory disease?**

① Yes ② No

1. **Did the frequency of respiratory infections decrease during the COVID-19 pandemic compared with the pre-COVID-19 pandemic?**

① Yes ② No

1. **The number of respiratory infections:**

( ) per year before the COVID-19 pandemic

( ) per year during the COVID-19 pandemic
